# An electrochemical sensor for detecting IL-6 expression following spinal cord injury

**DOI:** 10.1039/d5ra06654a

**Published:** 2025-11-27

**Authors:** Yuhan Jing, Chenxi Gu, Cai Wang, Bin Zhang

**Affiliations:** a Shandong First Medical University and Shandong Academy of Medical Sciences Jinan 250117 China; b Department of Orthopedic Surgery, The Affiliated Suzhou Hospital of Nanjing Medical University, Suzhou Municipal Hospital, Gusu School, Nanjing Medical University Suzhou China; c Binhai County People's Hospital Orthopedic Department Yancheng Jiangsu 224500 China xiaocaihong1027@163.com

## Abstract

Based on the principle of antigen-antibody specific binding, an electrochemical sensor utilizing Prussian blue nanoparticles (PBNPs) was developed to detect the expression of the inflammatory cytokine interleukin-6 (IL-6) *in vivo* following spinal cord injury (SCI). The high expression of pro-inflammatory cytokines following SCI contributes to numerous pathological processes in various diseases. Experimental results demonstrated that the prepared sensor exhibited advantages such as simplicity, high stability, reproducibility, high sensitivity, high specificity, and clinical applicability. Test results from animal models indicated that the inflammatory cytokine IL-6 was rapidly and highly expressed in rats following SCI. Furthermore, comparative analysis of clinical sample test results demonstrated that this sensor exhibited high accuracy. In summary, the prepared electrochemical sensor offered an exceptionally effective approach for investigating the expression trends of post-SCI inflammatory factors. It also holds broad prospects for translational clinical testing.

## Abstract 2

Based on the principle of antigen-antibody specific binding, an electrochemical sensor utilizing PBNPswas developed to detect the expression of IL-6 *in vivo* following SCI.

## Graphical abstract

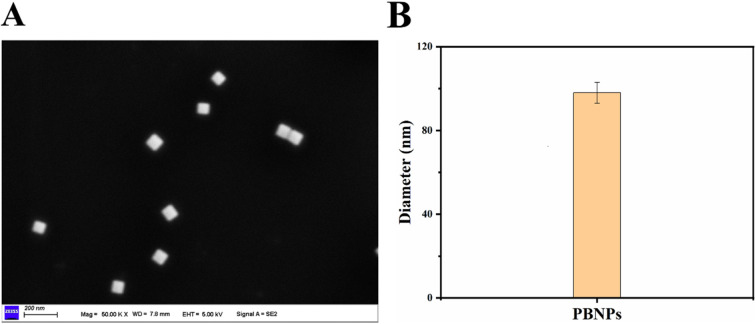

## 1 Introduction

Interleukin-6 (IL-6) is a multifunctional polypeptide cytokine, typically composed of two glycoprotein chains.^1^ During acute inflammatory responses, the body rapidly produces IL-6, which participates in and regulates the onset and progression of disease.^2^ Relevant studies indicate that IL-6 promotes the proliferation and differentiation of immune cells while inhibiting apoptosis.^3^ Furthermore, IL-6 plays a significant role in the onset, progression, and prognosis of tumors and certain metabolic diseases.^4^

Spinal cord injury (SCI) is a severe injury to the central nervous system.^5,6^ Following SCI, a series of inflammatory responses and secondary injuries occur.^7–9^ The expression of IL-6 surges dramatically during the acute phase of SCI, exerting a significant influence on the differentiation of neural stem cells.^10,11^ The expression of inflammatory mediators and the infiltration of immune cells constitute key components of the pathophysiological processes following SCI.^12^ Therefore, detecting the trend of IL-6 changes during the acute phase of SCI is crucial for understanding the pathological processes following SCI.

In recent years, numerous detection technologies had been developed for highly specific and sensitive detection of IL-6 expression *in vivo* and *in vitro*. Examples included enzyme-linked immunosorbent assays, photometric assays, SERS, and immunochromatographic assays.^13–18^ However, these methods suffer from low sensitivity, cumbersome operation, and high detection costs. A sensor based on SERS technology for detecting IL-6 has a detection limit of 0.5 pg mL^−1^. However, its preparation process is complex and relatively costly.^13^ Experiments have demonstrated that fibre-optic-based sensors can achieve spatially resolved detection of IL-6, offering high sensitivity, minimal sample requirements, and enabling *in vivo* monitoring. However, they cannot perform real-time monitoring and are highly dependent on high-end imaging equipment.^14^ The classic sandwich immunoassay method suffers from drawbacks such as dependence on equipment, limited testing environments, and lengthy detection times.^15,16^ Additionally, sensors combining microfluidic biochips with impedance cytometry have also been employed for IL-6 detection. While offering low cost and broad detection ranges, these methods suffer from low sensitivity, complex procedures, and significant variability.^17^ Label-free detection techniques based on field-effect transistors have also been developed, offering compact devices, high sensitivity, and rapid real-time monitoring. However, their application is constrained by limitations such as low specificity and sample processing requirements.^18^ In recent years, electrochemical sensors have gained extensive application in industrial and daily life due to their rapid response, high sensitivity, simplicity, and low cost, establishing themselves as one of the most promising detection technologies.^19–23^

**Scheme 1:**
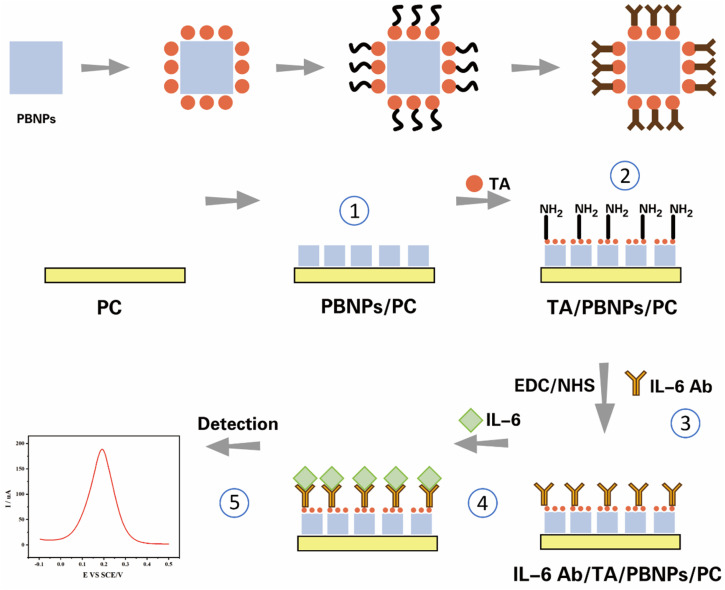
Schematic diagram of the preparation process and principle of electrochemical sensors.

In this study, to enable rapid, sensitive, and accurate detection of IL-6, we developed an electrochemical biosensor based on antigen-antibody specific interactions. First, Prussian blue nanoparticles (PBNPs) were modified onto the surface of a platinum-carbon electrode (PC). PBNPs serve as an excellent electrocatalytic material and endogenous redox probe, exhibiting a distinct, reversible redox peak during detection. The current intensity of this redox peak is highly sensitive to changes in the microenvironment at the electrode surface. Subsequently, the thionin acetate (TA), rich in amino groups on its surface, binds to the PBNPs, which are rich in carboxyl groups. The thiazine structure at the molecular centre of TA can be stably adsorbed onto the surface of PBNPs through strong π–π stacking and hydrophobic interactions, forming a robust molecular layer. Furthermore, TA provides abundant amino groups that enhance signal amplification and offer covalent attachment sites for subsequent antibody immobilisation. Simultaneously, TA itself functions as an electroactive dye. Its synergistic interaction with PBNPs enhances interfacial electron transport capabilities, amplifying electrochemical signals and thereby improving the sensor's sensitivity. To conjugate the IL-6 antibody to TA, (1-(3-dimethylaminopropyl)-3-ethylcarbodiimide hydrochloride/*N*-hydroxysuccinimide (EDC/NHS) was used to activate the surface amine groups on TA and the surface carboxyl groups on the antibody. The EDC/NHS mixture activates the surface carboxyl groups, generating reactive NHS esters. When the target molecule IL-6 binds specifically to immobilised antibodies, it forms a substantial, insulating protein layer (antibody-antigen immune complex) on the electrode surface (Scheme 1). The detection performance of the sensor was investigated using cyclic voltammetry (CV) and differential pulse voltammetry (DPV). In the presence of coexisting interferents at equal concentrations, including bovine serum albumin (BSA), interleukin-4 (IL-4), glycine (Gly), neuron-specific enolase (NSE), and sodium citrate (NaCit), the sensor demonstrated specific detection of IL-6. The sensor had an excellent linear detection range with a limit of detection (LOD) of 5.4 pg mL^−1^. Experimental results demonstrated that this electrochemical sensor offered advantages including rapid response, reproducibility, high stability, high sensitivity, and high specificity. Test results from SCI rats and sham-operated rats indicated that IL-6 expression significantly increased in rats during the acute phase of SCI. This sensor demonstrated exceptionally broad application prospects.

## 2 Materials

### 2.1 Materials and instruments

TA was purchased from Shanghai McLean Biochemical Technology Co., Ltd. Hydrochloric acid (HCl), sodium citrate (NaCit), potassium ferricyanide (K_3_[Fe(CN)_6_]), potassium chloride (KCl), bovine serum albumin (BSA), glutathione (GSH) and glycine (Gly) were all purchased from National Pharmaceutical Group Chemical Reagents Co., Ltd, USA. All purity levels exceed 99.9%. IL-6 antibodies (including anti-human and anti-rat), IL-6 antigens (including human and rat sources), IL-4 antigens (including human and rat sources), and neuron-specific enolase (NSE) were all procured from Sigma-Aldrich (Shanghai) Trading Co., Ltd. The electrochemical workstation (CHI660E), platinum-carbon electrode, reference electrode, and counter electrode were all purchased from Shanghai Chenhua Instruments Co., Ltd. The PB21 acidity meter was procured from Sediorius Scientific Instruments (Beijing) Co., Ltd. The scanning electron microscope employed for morphological characterisation of nanoparticles was the FE-SEM GeminiSEM 300 (Carl Zeiss, Germany). Particle size distribution was recorded using the NanoBrook 90Plus PALS electrostatic particle size analyser (Brookhaven Instruments, USA). ELISA results were assessed and calculated using a microplate reader (SpectraMax M5), procured from Meigu Molecular Instrument Co., Ltd (Shanghai, China). The grinding and polishing system was purchased from Ruiwo De Instruments Co., Ltd. Ultra-pure water filtration was performed using the Milli-Q reagent water system during the experiment.

### 2.2 Preparation of PBNPs

A 100 mL beaker was rinsed three times with detergent and three times with ultrapure water. Polyvinylpyrrolidone (PVP) (8 g), potassium ferricyanide (K_3_[Fe(CN)_6_]) (658 mg) and 40 mL hydrochloric acid (HCl) were mixed under magnetic stirring. The mixture was stirred for 2 h before being incubated in an 80 °C oven for 24 h. Following multiple washings and centrifugation, Prussian blue nanoparticles (PBNPs) were obtained.

### 2.3 Construction of electrochemical sensors

Following the preparation of PBNPs on the PC electrode, TA was subsequently modified onto the electrode surface to amplify the electrochemical signals of the modified electrode. The TA surface is rich in amino groups. To enhance the binding affinity between TA and the IL-6 antibody, we employed EDC/NHS to activate the amino groups on TA. Following thorough activation, the IL-6 antibody was immobilised onto the TA-modified electrode, ultimately yielding an electrochemical sensor. All the above reaction procedures were carried out in a constant-temperature incubator at 37 °C.

### 2.4 Detection of IL-6 in standard samples

When preparing standard solutions of IL-6 antigen at known concentrations, employ 0.1 M phosphate-buffered saline (PBS) as the solvent. Subsequently, add this to a mixture containing 0.005 M potassium ferricyanide (K_3_[Fe(CN)_6_]) and 0.1 M potassium chloride (KCl) (the reaction system solution). The constructed electrochemical sensor electrode was placed in the mixture, with the reference electrode and counter electrode simultaneously introduced. The experiment employed DPV detection, establishing a linear relationship between concentration and peak current, and calculating the sensor's detection limit.

### 2.5 Construction of the rat SCI model

The experimental animals were SPF-grade adult male SD rats (approximately 250 g, 8 weeks old), supplied by Jinan Pengyue Laboratory Animal Centre. All animal experiments were approved by the Institutional Animal Care and Use Committee of Shandong First Medical University and conducted in accordance with the National Institutes of Health Guidelines for the Care and Use of Laboratory Animals. The hair was removed from the tenth thoracic vertebra region of the rat. Povidone-iodine was used to disinfect the surgical site. Using a scalpel, the skin was incised to sequentially expose the subcutaneous tissue, muscles, and laminae. A burr was employed to open the spinal canal at the T9-T11 vertebral level in the rat, thereby fully exposing the spinal cord. Transfer the rats to the impact platform, secure them by their dorsal and ventral spinous processes, configure the impact apparatus parameters (depth = 1.0 mm, velocity = 2.0 m s^−1^, dwell time = 150 ms), localise the impact site, and deliver the impact to the exposed spinal cord. Rats not subjected to impact constitute the sham-operated group (Sham). Following successful establishment of the SCI model, blood samples were collected from the rats' tail veins and centrifuged to obtain the supernatant for subsequent analysis. All clinical samples were obtained following the patients' signing of informed consent forms. This study has been approved by the Ethics Committee of the Second Affiliated Hospital of Shandong First Medical University. A total of five clinical samples from patients with SCI were collected. After centrifugation to separate serum, the samples were stored frozen at −80 °C.

## 3 Results

### 3.1 Characterisation of PBNPs

**Fig. 1:**
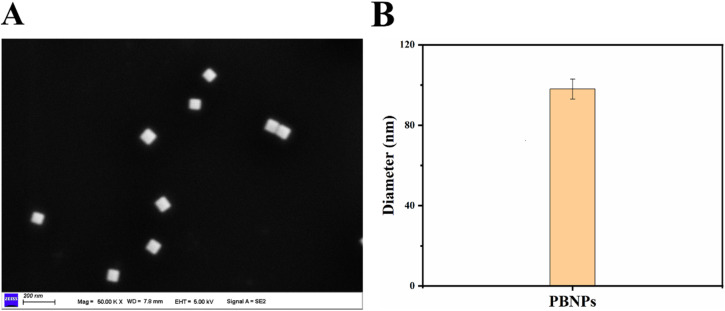
Characterisation of PBNPs. (A) SEM images of PBNPs. (B) Particle size of PBNPs.

To characterise the physical properties of PBNPs, we observed the morphology of the nanoparticles using scanning electron microscopy (SEM). As shown in Fig. 1A, PBNPs showed a tetrahedral morphology with stable and uniform structure. Furthermore, the particle size measurements of the three PBNPs were analysed and detected using a PALS electrostatic particle size analyser. The results indicated that the particle size of PBNPs was approximately 98 nm (Fig. 1B). Experimental findings demonstrated that the prepared PBNPs exhibited stable morphology and uniform particle size distribution.

### 3.2 Preparation of sensors

**Fig. 2:**
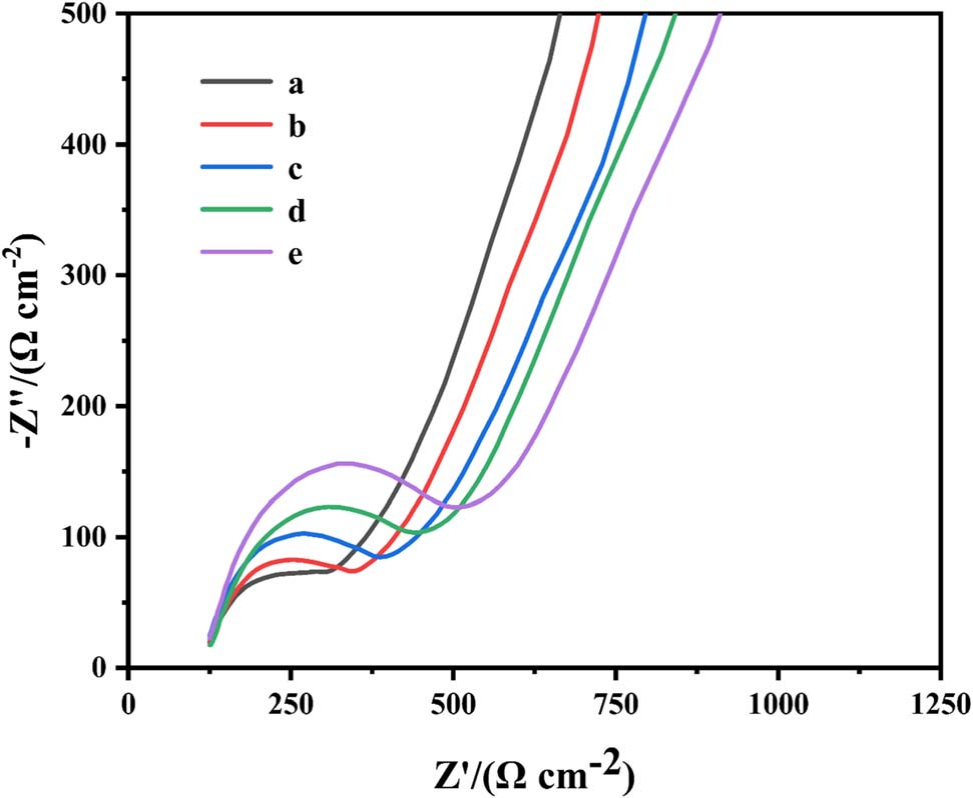
Electrochemical impedance spectroscopy of the sensor modification process.

To verify the completion of the IL-6 electrochemical sensor modification process, electrochemical impedance spectroscopy (EIS) measurements were performed on the electrochemical sensors at different modification stages. Electrochemical impedance spectroscopy (EIS) is a sensitive technique for tracking the electron transfer dynamics at interfaces. The electron transfer resistance (*R*_ct_) in its Nyquist plot is directly related to the insulating properties of the electrode surface. A higher *R*_ct_ value corresponds to a larger semicircle diameter, indicating greater resistance to electron transfer across the electrode/solution interface.^19^ Following modification of different substances, the EIS curves of the electrochemical sensors exhibited shifts, indicating alterations in the electrode's resistance values. As the modification reaction proceeded, the EIS curves for (a) PC, (b) PBNPs/PC, (c) TA/PBNPs/PC, (d) IL-6 Ab/TA/PBNPs/PC, and (e) IL-6/IL-6 Ab/TA/PBNPs/PC successively shifted upwards and to the right. The systematic, stepwise shift of the EIS spectrum from the lower left to the upper right perfectly aligns with the anticipated construction process of the sensing interface transitioning from “conductive” to “insulating”. This provides compelling electrochemical evidence for the successful completion of each modification step and the ultimate achievement of specific immune recognition. An increase in resistance value indicated the completion of the electrode surface modification process. To perform quantitative analysis of EIS data, we employed a suitably modified Randles equivalent circuit model, utilising ZSimpWin software to conduct a nonlinear least-squares fit of the experimental data (Fig. 2). We focused on analysing and comparing the *R*_ct_ fitting values of electrodes at different modification stages. The equivalent circuit comprises the solution resistance, constant phase element, electron transfer resistance, and Warburg impedance. The impedance is defined as ZCPE = 1/[*Q*(*jω*)*n*], where *Q* is the CPE constant and *n* is the dissipation factor (with values ranging from 0 ≤ *n* ≤ 1).

### 3.3 Sensor characteristics

**Fig. 3:**
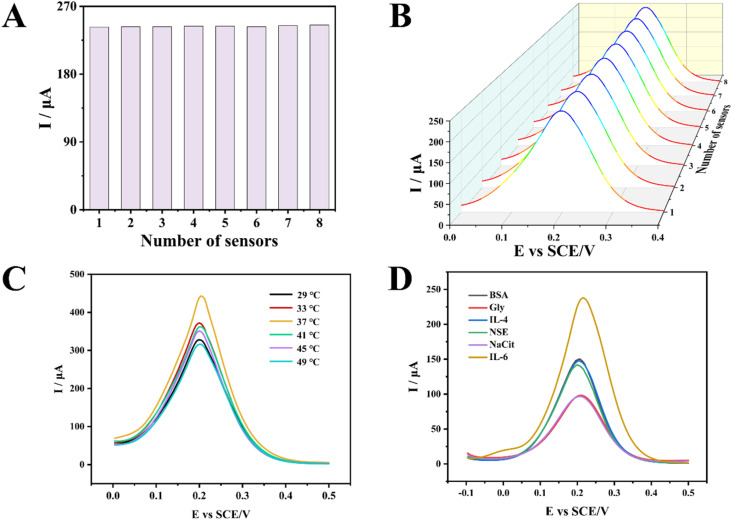
Characteristics of electrochemical sensors. (A) Bar chart of oxidation peak currents from eight DPV tests. (B) Three-dimensional map of DPV detection results from eight probes. (C) Peak current of DPV under different temperature conditions. (D) Sensor detection results for different substances.

Stability and repeatability are key indicators for evaluating sensor detection performance. Superior stability and repeatability can effectively enhance efficiency and reduce costs. A bar chart (Fig. 3A) based on the oxidation peak current values from eight DPV tests. Test results indicates that there was no significant difference in the oxidation peak current among the DPV measurements from the eight sensors. To validate the stability and repeatability of this sensor, we performed DPV measurements on eight sensors and plotted 3D maps (Fig. 3B). From the 3D waveform plots, the waveforms of the eight test results showed no significant variation, indicating excellent consistency in the sensor's waveforms. This demonstrated the sensor's good reproducibility. It holds broad application prospects in clinical sample testing. These findings demonstrated that the constructed IL-6 electrochemical sensor had excellent stability and reproducibility, thereby facilitating cost savings and enhanced efficiency. Additionally, to investigate the optimal operating temperature of the sensor, measurements were taken of an IL-6 solution at a concentration of 10 pg mL^−1^ while varying only the ambient temperature of the detection environment. As shown in Fig. 3C, the peak current of DPV varies with the ambient temperature of the reaction solution. It was noteworthy that the peak current reached its maximum value at a solution temperature of 37 °C. When the temperature was either higher or lower than 37 °C, the peak current decreased significantly. Experimental results indicated that the optimal operating temperature for the sensor is approximately 37 °C. This is closely aligned with the body temperature of clinical patients, holding significant implications for clinical translation. To verify the detection specificity of the sensor, BSA, Gly, IL-4, NSE, NaCit, and IL-6 were added to the reaction solution for testing(Fig. 3D). The concentrations of these substances were consistent at 10 pg mL^−1^. All other reaction conditions remained unchanged, such as temperature and pH. Experimental results indicated that the solution containing IL-6 exhibited the highest DPV detection peak. This demonstrated that the sensor possesses a degree of specificity in detecting IL-6. The experimental results above demonstrated that the fabricated sensor had favourable repeatability, stability, specificity, and practicality.

### 3.4 Kinetic properties of electrode sensors

**Fig. 4:**
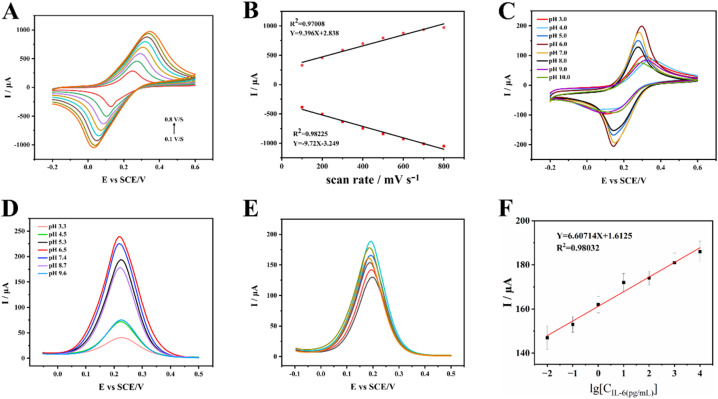
Kinetic properties of electrode sensors, suitable pH environment for sensor operation and results of the sample test. (A) CV detection results at different scanning rates. (B) Linear fitting relationship between redox current peaks and scanning rate. (C) CV detection results of the electrochemical sensor under different pH conditions. (D) DPV detection results of the electrochemical sensor under different pH conditions. (E) DPV detection results for different known concentrations of IL-6. (F) Linear relationship between oxidation peak current values and the logarithm of IL-6 concentration.

To evaluate the kinetic properties of the sensor, we recorded the CV curves of the sensor electrode at different scan rates (Fig. 4A). As the scanning rate increased from 0.1 V s^−1^ to 0.8 V s^−1^, the redox current at the electrode steadily increased. The peak potential continuously shifted with changes in scan rate. This indicated that the redox state of the electrochemical sensor was reversibly cycled, with the redox center located on the electrode surface. As the scan rate increases, the oxidation peak potential (*E*_pa_) shifts positively while the reduction peak potential (*E*_pc_) shifts negatively, causing the peak potential difference (Δ*E*_p_) to gradually increase. This indicates that it is a quasi-reversible process. As shown in Fig. 4B, we obtained a linear fitting relationship between the redox peak current and the scan rate through calculation. This excellent linear relationship indicated that the electrochemical reaction process at the electrode of this electrochemical sensor was a surface-controlled process.

To investigate the optimum operating pH for electrochemical sensors, CV and DPV measurements were conducted in reaction solutions at various pH values. As shown in Fig. 4C, the redox peak current value also varied with changes in the pH of the reaction solution. The detection results indicate that when the reaction solution was acidic or alkaline, the redox peak current value of the sensor was smaller. When the reaction solution has a pH of 6.0 or 7.0, the redox peak current value of the sensor increased significantly. This indicated that the sensor exhibits superior detection performance in neutral to slightly acidic reaction solution systems. Compared with the CV detection results, the DPV detection results (Fig. 4D) were more pronounced. In reaction solutions with pH values of 6.5 or 7.4, the peak currents were larger. Combining the results from CV and DPV measurements revealeed that the IL-6 electrochemical sensor performs optimally in a slightly acidic to neutral solution environment when detecting target IL-6. This indicated that pH 7.4 was most favourable for maintaining high-affinity binding between the IL-6 antibody and antigen. The influence of solution pH on sensor performance manifests primarily in two aspects: the stability of the electroactive material (PBNPs) and the efficiency of the antibody-antigen immune reaction. When the solution is acidic (pH < 6.0) or alkaline (pH > 8.0), the redox peak current of the CV is significantly attenuated. This occurs because PBNPs undergoes chemical decomposition under these conditions, leading to a gradual loss of electroactivity.^24^

To evaluate the electrochemical analytical performance of the IL-6 sensor, we detected IL-6 under the aforementioned optimised conditions. IL-6 was detected and analysed using DPV. Within the concentration range of 1 × 10^−3^ pg mL^−1^ to 1 × 10^3^ pg mL^−1^, the DPV oxidation peak current value of the IL-6 electrochemical sensor increased with rising IL-6 concentration (Fig. 4E). As shown in Fig. 4F, within the concentration range of 1 × 10^−3^ pg mL^−1^ to 1 × 10^3^ pg mL^−1^, the sensor's oxidation peak current value showed a good linear relationship with IL-6 concentration (*Y* = 6.60714*X* + 1.6125, *R*^2^ = 0.98032). Based on the aforementioned relationship, preliminary calculations indicate that the limit of detection (LOD) for this sensor was 5.4 pg mL^−1^. The detection limit was determined using a standard method. We measured the blank solution multiple times to obtain the standard deviation (*σ*) of its signal, and calculated the LOD according to the formula LOD = 3.3*σ*/*S* (where *S* is the slope of the calibration curve).

### 3.5 Animal sample testing

**Fig. 5:**
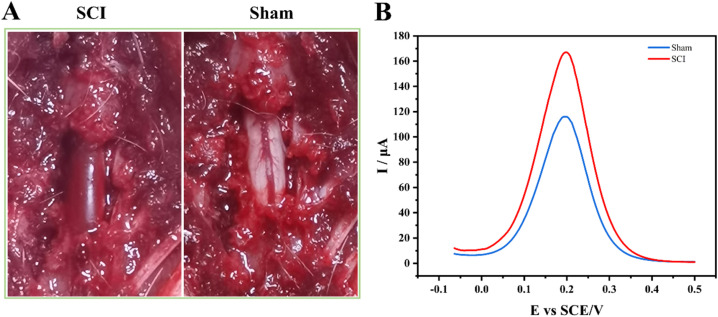
Animal model construction and testing. (A) Surgical images of animal model construction. (B) Test results for rats in the SCI and Sham groups.

To further evaluate the sensor's potential for use in clinical real-world sample testing, serum samples from SCI and Sham group rats were analysed using the sensor. Surgical images of rats in the Sham and SCI groups were shown in Fig. 5A. Rats in the SCI group marked spinal cord congestion and oedema. IL-6 expression in rat blood was measured 12 h post-spinal cord injury. Test results indicated that serum samples from SCI rats significantly higher IL-6 expression compared to Sham rats (Fig. 5B). IL-6 levels in the SCI group were significantly elevated by approximately 43.1% compared to the Sham group (*p* < 0.05). Experimental findings demonstrated that IL-6 expression rapidly increases in rats during the acute phase of SCI.

### 3.6 Clinical sample testing

To further validate the clinical applicability and accuracy of the sensor, the same clinical samples were tested using both enzyme-linked immunosorbent assay (ELISA) and the sensor. The detection results of both methods were shown in Table 1. The experimental results indicated that there was no significant difference between the sensor's detection results and those obtained *via* ELISA. This indicated that the sensor possesses excellent clinical applicability and accuracy, making it suitable for testing clinical samples.

**Table 1:** The sensor's detection results and the ELISA detection results

No.	ELISA (ng mL^−1^)	Sensor (ng mL^−1^)
1	62.80 ± 0.35	58.16 ± 0.52
2	117.63 ± 0.14	115.08 ± 0.32
3	143.34 ± 0.37	141.52 ± 0.61
4	277.68 ± 0.33	275.40 ± 0.26
5	398.35 ± 0.27	397.87 ± 0.34

## 4 Conclusion

We prepared a novel electrochemical sensor for detecting IL-6 by sequentially modifying the electrode surface with PBNPs, TA, and IL-6 antibodies. To gain a comprehensive understanding of the sensor's performance and operating conditions, we conducted tests using methods such as EIS, CV, and DPV. The results demonstrated that this electrochemical sensor had excellent detection performance characterized by high specificity, high sensitivity, high stability, and high reproducibility. The concentration of the DPV oxidation peak showed a good linear relationship with IL-6 concentration. The sensor's LOD was 5.4 pg mL^−1^. Based on the test results from animal and clinical samples, the prepared electrochemical sensors demonstrate significant potential for application.

## Ethics statement

The animal study was reviewed and approved by the Shandong first medical university. All clinical samples were obtained with informed consent signed by the patients. This study strictly adheres to the relevant laws, regulations, and ethical guidelines of the People's Republic of China and has received full approval from the Ethics Committee of the Second Affiliated Hospital of Shandong First Medical University (Ethics approval number: R202404150221).

## Author contributions

All authors listed have made a substantial, direct, and intellectual contribution to the work and approved it for publication. Bin Zhang: Revise the manuscript and reply to the reviewers' comments; Yuhan Jing: methodology, data Analysis; Chenxi Gu: writing – original Draft, collect clinical samples; Cai Wang: writing – review and editing.

## Conflicts of interest

The authors declare that the research was conducted in the absence of any commercial or financial relationships that could be construed as a potential conflict of interest.

## Data Availability

The raw data supporting the conclusion of this article will be made available by the authors, without undue reservation.
